# Supplementary material for: Diagnostic performance of a biotin-labeled 4D1 sandwich ELISA for serum antigen detection in talaromycosis
Source: PLoS Negl Trop Dis. 2026 May 5;20(5):e0014314. doi: 10.1371/journal.pntd.0014314 (PMC13167033; doi:10.1371/journal.pntd.0014314)
Supplement: S1 File — Detailed clinical characteristics of patients with culture-confirmed T. marneffei infection. Table B. Detailed microbiological findings and clinical characteristics of T. marneffei-negative patients with other microbial infections. Table C. Optimization of capture and detection antibody conditions for the sandwich ELISA. Table D. Optimization of streptavidin-HRP dilution in the sandwich ELISA. Table E. Optimization of serum dilution for antigen detection in the sandwich ELISA. Fig A. Biotinylation strategy showing NHS-ester activation and subsequent conjugation of avidin/streptavidin to primary amine-containing proteins. Fig B. Purification of mAb 4D1. Fig C. Controls validating the specificity of mAb 4D1 and its biotin-labeled conjugate in Western blot detection of TMCYA. Fig D. Optimization of capture and detection antibody conditions for the sandwich ELISA. Fig E. Optimization of streptavidin-HRP dilution in the sandwich ELISA. Fig F. Optimization of serum dilution for antigen detection in the sandwich ELISA. Fig G. Determination of analytical performance parameters (LOB, LOD, and LOQ) of the biotin-labeled 4D1 sandwich ELISA. (DOCX) [file pntd.0014314.s001.docx]

**Supplementary Materials**

The supplementary materials provide detailed clinical data and experimental procedures supporting the development and validation of the sandwich ELISA for *T. marneffei* antigen detection.

Tables A and Bsummarize the clinical and microbiological characteristics of patients included in this study. Table A presents detailed information on culture-confirmed *T. marneffei* cases, including demographic data, CD4⁺ T-cell counts, specimen types, and corresponding ELISA results. Table B includes patients with non-talaromycosis infections, providing a comprehensive overview of conditions used to evaluate assay specificity.

Tables C-Edescribe the stepwise optimization of key assay parameters. Table Cshows the optimization of capture antibody concentration and biotin-labeled 4D1 dilution. Table D presents the optimization of streptavidin-HRP dilution, and Table E summarizes the determination of the optimal serum dilution. These steps were performed sequentially to maximize signal-to-background separation, primarily evaluated using the P/N ratio and ΔOD values.

Figs A-Cpresent the structural basis and validation of the biotin-labeled monoclonal antibody (4D1). Fig A illustrates the biotinylation strategy based on NHS-ester activation and its conjugation to primary amine-containing proteins. Fig B shows the purification and characterization of mAb 4D1, including SDS-PAGE and Western blot analyses. Fig Cprovides control experiments validating the specificity of mAb 4D1 and its biotin-labeled conjugate.

Figs D-Fillustrate the optimization process. Fig Ddemonstrates the selection of optimal capture and detection antibody conditions. Fig Eshows the optimization of streptavidin-HRP dilution under fixed antibody conditions, and Fig Fpresents the determination of the optimal serum dilution.

Fig G illustrates the determination of analytical performance parameters, including the limit of blank (LOB), limit of detection (LOD), and limit of quantification (LOQ).

Overall, the supplementary data provide comprehensive methodological details and supporting evidence for assay performance, ensuring transparency and reproducibility.

**Table A. Detailed clinical characteristics of patients with culture-confirmed *T. marneffei infection* (n = 79)**

| **No.** | **Sex** | **Age** | **CD4⁺ T-cell count** (**cells/mm³**) | **Specimen test by in-house ELISA** | **In-house ELISA result (Positive/Negative)** | **Type of clinical specimen for diagnostic confirmation** | **Microorganism**  **(Identification)** |
| --- | --- | --- | --- | --- | --- | --- | --- |
| 1 | Female | 44 | 190 | Serum | Positive | Blood | *T. marneffei* |
| 2 | Male | 56 | 151 | Serum | Positive | Blood | *T. marneffei* |
| 3 | Female | 42 | 57 | Serum | Positive | Blood | *T. marneffei* |
| 4 | Male | 48 | 13 | Serum | Positive | Blood | *T. marneffei* |
| 5 | Female | 65 | NA^a^ | Serum | Positive | Blood | *T. marneffei* |
| 6 | Male | 44 | NA | Serum | Positive | Blood | *T. marneffei* |
| 7 | Male | 39 | NA | Serum | Positive | Blood | *T. marneffei* |
| 8 | Male | 35 | 8 | Serum | Positive | Blood | *T. marneffei* |
| 9 | Female | 61 | 165 | Serum | Negative | Blood | *T. marneffei* |
| 10 | Male | 50 | NA | Serum | Positive | Blood | *T. marneffei* |
| 11 | Male | 45 | NA | Serum | Positive | Blood | *T. marneffei* |
| 12 | Male | 49 | 15 | Serum | Positive | BALF^b^ | *T. marneffei*; *Candida albicans* |
| 13 | Male | 32 | 68 | Serum | Positive | Blood | *T. marneffei* |
| 14 | Male | 27 | 4 | Serum | Positive | Blood | *T. marneffei* |
| 15 | Male | 62 | 150 | Serum | Negative | Blood | *T. marneffei* |
| 16 | Female | 52 | NA | Serum | Positive | Blood | *T. marneffei* |

| **No.** | **Sex** | **Age** | **CD4⁺ T-cell count** (**cells/mm³**) | **Specimen test by in-house ELISA** | **In-house ELISA result (Positive/Negative)** | **Type of clinical specimen for diagnostic confirmation** | **Microorganism**  **(Identification)** |
| --- | --- | --- | --- | --- | --- | --- | --- |
| 17 | Male | 57 | 3 | Serum | Positive | Blood | *T. marneffei* |
| 18 | Female | 49 | 74 | Serum | Positive | Blood | *T. marneffei* |
| 19 | Male | 26 | 20 | Serum | Positive | Blood | *T. marneffei* |
| 20 | Female | 58 | NA | Serum | Positive | Blood | *T. marneffei* |
| 21 | Male | 24 | NA | Serum | Positive | Blood | *T. marneffei* |
| 22 | Male | 31 | NA | Serum | Negative | Blood | *T. marneffei* |
| 23 | Male | 30 | NA | Serum | Positive | Blood | *T. marneffei* |
| 24 | Male | 30 | NA | Serum | Positive | Blood | *T. marneffei* |
| 25 | Female | 20 | NA | Serum | Positive | Blood | *T. marneffei* |
| 26 | Male | 37 | NA | Serum | Positive | Blood | *T. marneffei* |
| 27 | Male | 65 | NA | Serum | Positive | Blood | *T. marneffei* |
| 28 | Female | 14 | 4 | Serum | Positive | Blood | *T. marneffei* |
| 29 | Female | 43 | NA | Serum | Positive | Blood | *T. marneffei* |
| 30 | Female | 43 | NA | Serum | Positive | Blood | *T. marneffei* |
| 31 | Male | 29 | 16 | Serum | Positive | Blood | *T. marneffei* |
| 32 | Female | 16 | NA | Serum | Positive | Blood | *T. marneffei* |
| 33 | Female | 14 | 1 | Serum | Positive | Blood | *T. marneffei* |

| **No.** | **Sex** | **Age** | **CD4⁺ T-cell count** (**cells/mm³**) | **Specimen test by in-house ELISA** | **In-house ELISA result (Positive/Negative)** | **Type of clinical specimen for diagnostic confirmation** | **Microorganism**  **(Identification)** |
| --- | --- | --- | --- | --- | --- | --- | --- |
| 34 | Male | 8 | NA | Serum | Negative | Blood | *T. marneffei* |
| 35 | Female | 38 | 9 | Serum | Positive | Blood | *T. marneffei* |
| 36 | Male | 37 | 1 | Serum | Positive | Blood | *T. marneffei* |
| 37 | Male | 34 | NA | Serum | Positive | Blood | *T. marneffei* |
| 38 | Male | 28 | NA | Serum | Negative | Blood | *T. marneffei* |
| 39 | Female | 21 | NA | Serum | Positive | Blood | *T. marneffei* |
| 40 | Male | 25 | NA | Serum | Negative | Blood | *T. marneffei* |
| 41 | Male | 1 | NA | Serum | Positive | Blood | *T. marneffei* |
| 42 | Male | 45 | NA | Serum | Positive | Blood | *T. marneffei* |
| 43 | Female | 41 | NA | Serum | Positive | Blood | *T. marneffei* |
| 44 | Female | 43 | 6 | Serum | Positive | Blood | *T. marneffei* |
| 45 | Female | 34 | NA | Serum | Positive | Blood | *T. marneffei* |
| 46 | Male | 26 | 20 | Serum | Positive | Blood | *T. marneffei* |
| 47 | Male | 24 | 8 | Serum | Positive | Blood | *T. marneffei* |
| 48 | Male | 23 | NA | Serum | Positive | Blood | *T. marneffei* |
| 49 | Male | 30 | NA | Serum | Positive | Blood | *T. marneffei* |
| 50 | Female | 58 | NA | Serum | Positive | Blood | *T. marneffei* |

| **No.** | **Sex** | **Age** | **CD4⁺ T-cell count** (**cells/mm³**) | **Specimen test by in-house ELISA** | **In-house ELISA result (Positive/Negative)** | **Type of clinical specimen for diagnostic confirmation** | **Microorganism**  **(Identification)** |
| --- | --- | --- | --- | --- | --- | --- | --- |
| 51 | Male | 58 | NA | Serum | Positive | Tissue biopsy | *T. marneffei* |
| 52 | Male | 40 | NA | Serum | Positive | BALF; Bone marrow; Blood | *T. marneffei*; *Candida albicans* |
| 53 | Male | 31 | 7 | Serum | Positive | Blood | *T. marneffei* |
| 54 | Male | 31 | NA | Serum | Positive | Blood | *T. marneffei* |
| 55 | Male | 52 | NA | Serum | Positive | Skin biopsy | *T. marneffei* |
| 56 | Male | 24 | 3 | Serum | Positive | Blood | *T. marneffei* |
| 57 | Male | 25 | 136 | Serum | Positive | Tissue biopsy | *T. marneffei* |
| 58 | Male | 28 | 6 | Serum | Positive | Blood | *T. marneffei* |
| 59 | Male | 26 | 102 | Serum | Positive | Blood | *T. marneffei* |
| 60 | Male | 44 | 4 | Serum | Positive | Blood | *T. marneffei* |
| 61 | Male | 29 | NA | Serum | Negative | Blood | *T. marneffei* |
| 62 | Male | 44 | 14 | Serum | Positive | Blood | *T. marneffei* |
| 63 | Female | 59 | 20 | Serum | Positive | Blood | *T. marneffei* |
| 64 | Female | 41 | 15 | Serum | Positive | Blood | *T. marneffei* |
| 65 | Male | 56 | 6 | Serum | Positive | Blood | *T. marneffei* |
| 66 | Male | 39 | 17 | Serum | Positive | Blood | *T. marneffei* |

| **No.** | **Sex** | **Age** | **CD4⁺ T-cell count** (**cells/mm³**) | **Specimen test by in-house ELISA** | **In-house ELISA result (Positive/Negative)** | **Type of clinical specimen for diagnostic confirmation** | **Microorganism**  **(Identification)** |
| --- | --- | --- | --- | --- | --- | --- | --- |
| 67 | Male | 70 | 103 | Serum | Positive | Blood | *T. marneffei* |
| 68 | Female | 52 | 19 | Serum | Positive | Blood | *T. marneffei* |
| 69 | Male | 39 | 27 | Serum | Positive | Blood | *T. marneffei* |
| 70 | Male | 33 | 19 | Serum | Positive | Blood | *T. marneffei* |
| 71 | Female | 74 | 36 | Serum | Negative | Blood | *T. marneffei* |
| 72 | Female | 70 | NA | Serum | Positive | Blood | *T. marneffei* |
| 73 | Male | 44 | NA | Serum | Positive | Blood | *T. marneffei* |
| 74 | Male | 29 | 2 | Serum | Positive | Blood | *T. marneffei* |
| 75 | Male | 47 | 6 | Serum | Positive | Blood | *T. marneffei* |
| 76 | Male | 37 | 1 | Serum | Positive | Blood | *T. marneffei* |
| 77 | Female | 51 | 28 | Serum | Negative | Skin biopsy | *T. marneffei* |
| 78 | Male | 43 | 4 | Serum | Positive | Blood | *T. marneffei* |
| 79 | Female | 45 | 1 | Serum | Positive | Blood | *T. marneffei* |

**Note:** a, NA, not available; b, BALF, bronchoalveolar lavage fluid. Positive *T. marneffei* cultures were confirmed by thermal dimorphic transition between 25°C and 37°C and microscopic analysis. In-house ELISA results were interpreted based on optical density (OD) values; an OD cut-off of 0.268, determined by receiver operating characteristic (ROC) curve analysis, was used to classify results as positive (> 0.268) or negative (≤ 0.268).

**Table B. Detailed microbiological findings and clinical characteristics of *T. marneffei*-negative patients with other microbial infections (n = 139)**

| **No.** | **Sex** | **Age** | **CD4⁺ T-cell count** (**cells/mm³**) | **Specimen test by in-house ELISA** | **In-house ELISA result (Positive/Negative)** | **Type of clinical specimen for diagnostic confirmation** | **Microorganism**  **(Identification)** |
| --- | --- | --- | --- | --- | --- | --- | --- |
| 1 | Male | 65 | NA^a^ | Serum | Negative | Sputum | Staphylococcus spp.; Streptococcus spp. |
| 2 | Female | 24 | 354 | Serum | Negative | Blood | HCV^c^ |
| 3 | Male | 70 | 2 | Serum | Negative | Sputum; BALF^b^; Blood | Staphylococcus aureus; mixed Gram-negative bacilli; unidentified dematiaceous fungi; CMV^d^ |
| 4 | Male | 60 | 26 | Serum | Negative | Blood | HBV^e^ |
| 5 | Male | 69 | 83 | Serum | Negative | Skin(scrotum); Urine; Sputum | *Escherichia coli* |
| 6 | Female | 82 | NA | Serum | Negative | Sputum | *Enterococcus* spp.; *Staphylococcus* spp. |
| 7 | Female | 52 | 985 | Serum | Negative | CSF^f^; Stool; Blood | *Cryptococcus* spp.; *Enterococcus* spp.; *Escherichia coli*; EBV^g^ |
| 8 | Male | 31 | NA | Serum | Negative | Blood | Influenza B virus; EBV |
| 9 | Female | 68 | NA | Serum | Negative | Blood | *Candida* spp. |
| 10 | Female | 29 | 23 | Serum | Negative | BALF; Blood | *Mycobacterium* spp.; HBV |
| 11 | Female | 63 | NA | Serum | Negative | Blood; BALF | *Mycobacterium* spp.; *Streptococcus* spp. |
| 12 | Female | 63 | NA | Serum | Negative | Urine (unidentified species) | Mixed organisms |

| **No.** | **Sex** | **Age** | **CD4⁺ T-cell count** (**cells/mm³**) | **Specimen test by in-house ELISA** | **In-house ELISA result (Positive/Negative)** | **Type of clinical specimen for diagnostic confirmation** | **Microorganism**  **(Identification)** |
| --- | --- | --- | --- | --- | --- | --- | --- |
| 13 | Female | 55 | 1305 | Serum | Negative | Sputum | *Staphylococcus* spp.; *Streptococcus* spp. |
| 14 | Male | 55 | NA | Serum | Negative | Urine; Stool | *Candida* spp.; *Enterococcus* spp. |
| 15 | Female | 55 | NA | Serum | Negative | Bone marrow | *Streptococcus* spp. |
| 16 | Female | 68 | NA | Serum | Negative | Blood | *Candida* spp. |
| 17 | Male | 67 | NA | Serum | Negative | Sputum | *Streptococcus* spp. |
| 18 | Male | 38 | NA | Serum | Negative | Sputum | *Enterococcus* spp.; *Staphylococcus* spp. |
| 19 | Male | 52 | 339 | Serum | Negative | Sputum | *Enterococcus s*pp.; *Staphylococcus* spp. |
| 20 | Male | 54 | NA | Serum | Negative | BALF | *Mycobacterium* spp. |
| 21 | Male | 73 | 7 | Serum | Negative | Blood; Sputum | *Enterococcus* spp.; *Staphylococcus* spp. |
| 22 | Male | 51 | 33 | Serum | Negative | Stool | *Shigella flexneri* |
| 23 | Female | 21 | NA | Serum | Negative | Blood; Sputum; BALF | *Enterococcus* spp.; *Streptococcus* spp. |
| 24 | Female | 68 | NA | Serum | Positive | Sputum; Stool | *Enterococcus* spp. |
| 25 | Male | 60 | NA | Serum | Negative | BALF | *Staphylococcus* spp.; *Streptococcus* spp. |
| 26 | Male | 56 | NA | Serum | Negative | CSF | *Cryptococcus* spp. |
| 27 | Male | 54 | NA | Serum | Negative | Skin (left arm) | *Mycobacterium* spp. |
| 28 | Male | 62 | NA | Serum | Negative | Skin (chin) | Encapsulated budding yeast (*Cryptococcus*-like) |

| **No.** | **Sex** | **Age** | **CD4⁺ T-cell count** (**cells/mm³**) | **Specimen test by in-house ELISA** | **In-house ELISA result (Positive/Negative)** | **Type of clinical specimen for diagnostic confirmation** | **Microorganism**  **(Identification)** |
| --- | --- | --- | --- | --- | --- | --- | --- |
| 29 | Male | 62 | NA | Serum | Negative | Blood | *Staphylococcus spp.* |
| 30 | Male | 34 | 2 | Serum | Negative | Blood | *Coryneform bacteria* |
| 31 | Female | 19 | NA | Serum | Negative | CSF; Stool | *Cryptococcus* spp.; *Streptococcus* spp. |
| 32 | Female | 63 | NA | Serum | Negative | Tissue (Skin) | *Sporothrix schenckii; Staphylococcus* spp. |
| 33 | Male | 48 | 349 | Serum | Negative | Urine | *Escherichia coli* |
| 34 | Male | 61 | NA | Serum | Negative | BALF | *Aspergillus spp.* |
| 35 | Male | 56 | NA | Serum | Negative | CSF; Sputum; Stool | *Cryptococcus* spp.; *Enterococcus* spp. |
| 36 | Female | 68 | NA | Serum | Negative | Tissue (T6-T7) | *Nontuberculous mycobacteria* |
| 37 | Male | 25 | 192 | Serum | Negative | Sputum; Stool | *Enterococcus* spp. |
| 38 | Female | 57 | NA | Serum | Negative | Sputum | *Cryptococcus* spp. |
| 39 | Female | 14 | NA | Serum | Negative | Sputum | *Candida* spp.; *Cryptococcus* spp. |
| 40 | Male | 36 | 631 | Serum | Negative | Skin | *Nontuberculous mycobacteria* (NTM) |
| 41 | Male | 33 | NA | Serum | Negative | Sputum | *Streptococcus* spp. |
| 42 | Female | 56 | NA | Serum | Negative | Pus (Rt. knee fluid) | *Streptococcus* spp. |
| 43 | Female | 52 | NA | Serum | Negative | CSF | *Cryptococcus* spp. |
| 44 | Female | 36 | 921 | Serum | Negative | Blood | HBV; HCV |
| 45 | Female | 14 | 1027 | Serum | Negative | Sputum | *Candida* spp.; *Cryptococcus* spp. |

| **No.** | **Sex** | **Age** | **CD4⁺ T-cell count** (**cells/mm³**) | **Specimen test by in-house ELISA** | **In-house ELISA result (Positive/Negative)** | **Type of clinical specimen for diagnostic confirmation** | **Microorganism**  **(Identification)** |
| --- | --- | --- | --- | --- | --- | --- | --- |
| 46 | Female | 51 | 3080 | Serum | Negative | Blood; BALF | *Staphylococcus* spp.; *Streptococcus* spp. |
| 47 | Female | 14 | 1027 | Serum | Negative | Blood | *Cryptococcus* spp. |
| 48 | Male | 27 | NA | Serum | Negative | Blood | *Corynebacterium striatum* |
| 49 | Male | 91 | NA | Serum | Negative | Sputum | *Candida* spp. |
| 50 | Female | 69 | NA | Serum | Negative | Sputum (unidentified species) | Yeast; mixed Gram-negative bacilli |
| 51 | Female | 67 | NA | Serum | Negative | BALF | *Staphylococcus* spp. |
| 52 | Male | 54 | 5 | Serum | Negative | Sputum (unidentified fungus species) | Yeast |
| 53 | Male | 39 | NA | Serum | Negative | Blood; Sputum | *Mycobacterium* spp. |
| 54 | Female | 50 | NA | Serum | Negative | Sputum | *Enterococcus* spp.; *Staphylococcus* spp. |
| 55 | Male | 41 | 287 | Serum | Negative | Pus | *Escherichia coli* |
| 56 | Female | 26 | 94 | Serum | Negative | Blood | *Cryptococcus* spp. |
| 57 | Male | 63 | NA | Serum | Negative | Lymph node biopsy | *Streptococcus spp.* |
| 58 | Female | 71 | NA | Serum | Negative | Blood | *EBV* |
| 59 | Male | 27 | NA | Serum | Negative | Blood | *Cryptococcus spp.* |
| 60 | Male | 37 | 101 | Serum | Negative | CSF; Blood | *Mycobacterium* spp.; HBV |
| 61 | Male | 72 | NA | Serum | Negative | Sputum | *Cryptococcus* spp. |
| 62 | Male | 37 | 101 | Serum | Negative | CSF | *Mycobacterium* spp. |
| 63 | Female | 20 | NA | Serum | Negative | BALF (unidentified fungus species) | Hyaline fungi |

| **No.** | **Sex** | **Age** | **CD4⁺ T-cell count** (**cells/mm³**) | **Specimen test by in-house ELISA** | **In-house ELISA result (Positive/Negative)** | **Type of clinical specimen for diagnostic confirmation** | **Microorganism**  **(Identification)** |
| --- | --- | --- | --- | --- | --- | --- | --- |
| 64 | Male | 36 | 16 | Serum | Negative | Sputum | *Mycobacterium tuberculosis* |
| 65 | Male | 72 | NA | Serum | Negative | Blood; Sputum | *Cryptococcus* spp.; *Streptococcus* spp. |
| 66 | Male | 19 | 183 | Serum | Negative | Sputum | *Staphylococcus* spp. |
| 67 | Female | 15 | NA | Serum | Negative | CSF | *Cryptococcus spp.; Streptococcus spp.* |
| 68 | Male | 66 | NA | Serum | Negative | Tissue biopsy | *Dematiaceous fungi* |
| 69 | Male | 19 | 484 | Serum | Negative | Sputum | *Staphylococcus spp.; Streptococcus spp.* |
| 70 | Female | 76 | NA | Serum | Negative | Blood | *Cryptococcus spp.* |
| 71 | Female | 68 | NA | Serum | Negative | Blood; BALF | *Streptococcus* spp. |
| 72 | Male | 29 | 427 | Serum | Negative | Pus (from tissue) | *Mycobacterium* spp. |
| 73 | Male | 64 | 127 | Serum | Negative | Blood | *Cryptococcus* spp.; *Escherichia coli* |
| 74 | Male | 46 | NA | Serum | Negative | Sputum; Pus | *Mycobacterium tuberculosis* complex; *Klebsiella pneumoniae*; *Pseudomonas aeruginosa* (carbapenem-resistant) |
| 75 | Male | 68 | NA | Serum | Negative | Lymph node biopsy | *Histoplasma capsulatum* |
| 76 | Male | 36 | NA | Serum | Negative | Blood | *Cryptococcus* spp.; *Staphylococcus* spp. |
| 77 | Male | 45 | 542 | Serum | Negative | Blood | HBV; HCV |

| **No.** | **Sex** | **Age** | **CD4⁺ T-cell count** (**cells/mm³**) | **Specimen test by in-house ELISA** | **In-house ELISA result (Positive/Negative)** | **Type of clinical specimen for diagnostic confirmation** | **Microorganism**  **(Identification)** |
| --- | --- | --- | --- | --- | --- | --- | --- |
| 78 | Female | 26 | NA | Serum | Negative | Pus | *Streptococcus* spp. |
| 79 | Male | 46 | 103 | Serum | Negative | Blood | *Cryptococcus* spp. |
| 80 | Female | 97 | NA | Serum | Negative | BALF | *Candida* spp.; *Streptococcus* spp. |
| 81 | Female | 63 | NA | Serum | Negative | Tissue biopsy | *Cryptococcus* spp. |
| 82 | Female | 69 | NA | Serum | Positive | Blood | *Cryptococcus* spp. |
| 83 | Female | 20 | NA | Serum | Negative | BALF | *Streptococcus* spp. |
| 84 | Female | 20 | NA | Serum | Negative | Blood | *Cryptococcus* spp. |
| 85 | Male | 63 | 644 | Serum | Negative | Pus (tonsil); Blood | *Haemophilus parainfluenzae; Commensal Neisseria* spp.; Viridans group streptococci; EBV |
| 86 | Male | 39 | 116 | Serum | Negative | CSF | *Cryptococcus* spp. |
| 87 | Male | 29 | 59 | Serum | Negative | Blood | *Cryptococcus* spp. |
| 88 | Male | 56 | 175 | Serum | Negative | Stool | *Opisthorchis viverrini* |
| 89 | Female | 34 | NA | Serum | Negative | Tissue biopsy | *Phanerochaete* spp. |
| 90 | Male | 68 | NA | Serum | Negative | Blood | *Cryptococcus* spp. |
| 91 | Male | 58 | NA | Serum | Negative | Tissue biopsy | *Mycobacterium* spp. |
| 92 | Male | 68 | NA | Serum | Negative | Blood | *Cryptococcus* spp. |

| **No.** | **Sex** | **Age** | **CD4⁺ T-cell count** (**cells/mm³**) | **Specimen test by in-house ELISA** | **In-house ELISA result (Positive/Negative)** | **Type of clinical specimen for diagnostic confirmation** | **Microorganism**  **(Identification)** |
| --- | --- | --- | --- | --- | --- | --- | --- |
| 93 | Male | 27 | NA | Serum | Negative | Blood | *Cryptococcus* spp. |
| 94 | Male | 31 | 58 | Serum | Negative | Blood | HBV |
| 95 | Female | 59 | NA | Serum | Negative | Sputum | *Enterococcus* spp.; Mycobacterium spp.; *Staphylococcus* spp. |
| 96 | Male | 28 | 94 | Serum | Negative | Blood; Sputum | *Mycobacterium* spp. |
| 97 | Female | 47 | 141 | Serum | Negative | Blood | HBV |
| 98 | Male | 33 | 19 | Serum | Negative | Blood | *Toxoplasma gondii* |
| 99 | Female | 63 | 33 | Serum | Negative | BALF | *Candida* spp.; *Streptococcus* spp. |
| 100 | Male | 65 | NA | Serum | Negative | Sputum | *Staphylococcus* spp.; *Streptococcus* spp. |
| 101 | Male | 29 | NA | Serum | Negative | Tissue biopsy | *Mycobacterium* spp. |
| 102 | Male | 68 | NA | Serum | Negative | BALF | *Candida* spp.; *Staphylococcus* spp.; *Streptococcus* spp. |
| 103 | Male | 75 | NA | Serum | Negative | Sputum | *Candida* spp. |
| 104 | Male | 46 | 259 | Serum | Negative | Blood | HBV |
| 105 | Male | 60 | 311 | Serum | Negative | Blood | *Cryptococcus* spp. |
| 106 | Female | 88 | NA | Serum | Negative | Urine | *Candida* spp. |
| 107 | Male | 39 | 11 | Serum | Negative | Tissue biopsy; Blood | *Staphylococcus* spp.; *Streptococcus* spp.; HBV |
| 108 | Male | 50 | NA | Serum | Negative | Blood | *Candida* spp. |
| 109 | Female | 66 | NA | Serum | Negative | BALF | *Legionella* spp. |

| **No.** | **Sex** | **Age** | **CD4⁺ T-cell count** (**cells/mm³**) | **Specimen test by in-house ELISA** | **In-house ELISA result (Positive/Negative)** | **Type of clinical specimen for diagnostic confirmation** | **Microorganism**  **(Identification)** |
| --- | --- | --- | --- | --- | --- | --- | --- |
| 110 | Female | 53 | NA | Serum | Negative | Tissue biopsy (Skin) | *Mycobacterium* spp. |
| 111 | Male | 35 | 85 | Serum | Negative | Tissue (Colon) | *Candida* spp. |
| 112 | Male | 71 | NA | Serum | Negative | Lymph node biopsy | *Mycobacterium* spp. |
| 113 | Female | 69 | NA | Serum | Negative | Brain abscess | *Salmonella enterica* |
| 114 | Female | 22 | 457 | Serum | Positive | Pus; Blood | *Streptococcus* spp.; HBV |
| 115 | Male | 50 | 46 | Serum | Negative | Blood | HBV |
| 116 | Male | 50 | 47 | Serum | Negative | Blood | HBV |
| 117 | Male | 44 | NA | Serum | Negative | Blood | *Mycobacterium* spp. |
| 118 | Female | 23 | NA | Serum | Negative | Blood | EBV |
| 119 | Male | 21 | 454 | Serum | Negative | Pus | *Staphylococcus* spp.; Streptococcus spp. |
| 120 | Male | 34 | NA | Serum | Negative | BALF | *Cryptococcus* spp.; Mycobacterium spp. |
| 121 | Male | 24 | NA | Serum | Negative | Tissue biopsy (Skin) | *Streptococcus* spp. |
| 122 | Female | 64 | NA | Serum | Negative | BALF | *Mycobacterium tuberculosis* complex |
| 123 | Female | 68 | NA | Serum | Negative | Blood; Sputum | *Candida* spp. |
| 124 | Female | 48 | 9 | Serum | Negative | Blood | *Cryptococcus* spp. |
| 125 | Female | 15 | NA | Serum | Negative | Blood; CSF | *Cryptococcus* spp.; *Streptococcus* spp. |
| 126 | Male | 61 | NA | Serum | Negative | Sputum | *Nocardia* spp. |
| 127 | Female | 55 | NA | Serum | Negative | Blood | *Nontuberculous mycobacteria* (NTM) |

| **No.** | **Sex** | **Age** | **CD4⁺ T-cell count** (**cells/mm³**) | **Specimen test by in-house ELISA** | **In-house ELISA result (Positive/Negative)** | **Type of clinical specimen for diagnostic confirmation** | **Microorganism**  **(Identification)** |
| --- | --- | --- | --- | --- | --- | --- | --- |
| 128 | Female | 97 | NA | Serum | Negative | Blood | *Candida* spp. |
| 129 | Male | 57 | NA | Serum | Negative | Lymph node biopsy | *Nontuberculous mycobacteria* (NTM) |
| 130 | Female | 29 | 122 | Serum | Negative | Blood | *Cryptococcus* spp. |
| 131 | Male | 68 | NA | Serum | Negative | BALF | *Candida* spp. |
| 132 | Female | 15 | NA | Serum | Negative | CSF | *Cryptococcus* spp. |
| 133 | Male | 60 | NA | Serum | Negative | Blood | *Cryptococcus* spp. |
| 134 | Male | 69 | NA | Serum | Negative | Sputum | *Candida* spp. |
| 135 | Male | 78 | NA | Serum | Negative | BALF | *Candida* spp. |
| 136 | Male | 74 | NA | Serum | Negative | BALF | *Mycobacterium* spp. |
| 137 | Female | 40 | 28 | Serum | Negative | Blood | *Toxoplasma gondii* |
| 138 | Male | 29 | NA | Serum | Negative | CSF | *Streptococcus* spp. |
| 139 | Female | 63 | NA | Serum | Negative | BALF | *Mycobacterium* spp. |

## **Note:** a, NA, not available; b, BALF, bronchoalveolar lavage fluid; c, HCV, hepatitis C virus; d, CMV, cytomegalovirus; e, HBV, hepatitis B virus; f, CSF, cerebrospinal fluid; g, EBV, Epstein-Barr virus. Clinical specimens were identified by culture, molecular, serological, or microscopic methods. CD4⁺ T-cell counts were unavailable for some patients due to incomplete laboratory testing. In-house ELISA results were interpreted based on optical density (OD) values; an OD cut-off of 0.268, determined by receiver operating characteristic (ROC) curve analysis, was used to classify results as positive (> 0.268) or negative (≤ 0.268).

**Table C. Optimization of capture and detection antibody conditions for the sandwich ELISA**

| **Capture antibody (µg/well)** | **Biotin-labeled 4D1 (ratio)** | **Detection biotin-labeled 4D1 dilution** | | | | | | | | | | | | | | |
| --- | --- | --- | --- | --- | --- | --- | --- | --- | --- | --- | --- | --- | --- | --- | --- | --- |
|  |  | **1:10** | | | **1:100** | | | **1:1000** | | | **1:1500** | | | **1:2000** | | |
|  |  | **Mean OD ± SD (+)** | **Mean OD ± SD (-)** | **P/N ratio** | **Mean OD ± SD (+)** | **Mean OD ± SD (-)** | **P/N ratio** | **Mean OD ± SD (+)** | **Mean OD ± SD (-)** | **P/N ratio** | **Mean OD ± SD (+)** | **Mean OD ± SD (-)** | **P/N ratio** | **Mean OD ± SD (+)** | **Mean OD ± SD (-)** | **P/N ratio** |
| 0.1 | 5:1 | 0.221 ± 0.002 | 0.199 ± 0.002 | 1.112 | 0.200 ± 0.003 | 0.180 ± 0.002 | 1.115 | 0.201 ± 0.002 | 0.179 ± 0.002 | 1.123 | 0.190 ± 0.01 | 0.166 ± 0.001 | 1.143 | 0.191 ± 0.002 | 0.180 ± 0.002 | 1.061 |
|  | 10:1 | 0.202 ± 0.001 | 0.201 ± 0.002 | 1.003 | 0.206 ± 0.007 | 0.190 ± 0.010 | 1.088 | 0.200 ± 0.001 | 0.191 ± 0.002 | 1.049 | 0.221 ± 0.002 | 0.190 ± 0.002 | 1.161 | 0.199 ± 0.001 | 0.198 ± 0.001 | 1.005 |
|  | 20:1 | 0.257 ± 0.003 | 0.190 ± 0.010 | 1.355 | 0.260 ± 0.001 | 0.197 ± 0.006 | 1.318 | 0.300 ± 0.002 | 0.199 ± 0.002 | 1.508 | 0.260 ± 0.001 | 0.191 ± 0.002 | 1.359 | 0.212 ± 0.002 | 0.182 ± 0.006 | 1.161 |
|  | 30:1 | 0.246 ± 0.004 | 0.230 ± 0.001 | 1.068 | 0.239 ± 0.005 | 0.218 ± 0.005 | 1.096 | 0.250 ± 0.001 | 0.201 ± 0.002 | 1.244 | 0.230 ± 0.001 | 0.214 ± 0.012 | 1.075 | 0.218 ± 0.005 | 0.209 ± 0.004 | 1.043 |
|  | 40:1 | 0.222 ± 0.001 | 0.221 ± 0.002 | 1.006 | 0.200 ± 0.001 | 0.199 ± 0.001 | 1.005 | 0.221 ± 0.002 | 0.195 ± 0.005 | 1.134 | 0.202 ± 0.001 | 0.201 ± 0.001 | 1.007 | 0.231 ± 0.001 | 0.230 ± 0.007 | 1.004 |
|  | 50:1 | 0.214 ± 0.001 | 0.213 ± 0.004 | 1.002 | 0.206 ± 0.002 | 0.205 ± 0.002 | 1.008 | 0.214 ± 0.012 | 0.198 ± 0.002 | 1.079 | 0.200 ± 0.002 | 0.191 ± 0.002 | 1.047 | 0.211 ± 0.001 | 0.208 ± 0.007 | 1.016 |

| **Capture antibody (µg/well)** | **Biotin-labeled 4D1 (ratio)** | **Detection biotin-labeled 4D1 dilution** | | | | | | | | | | | | | | |
| --- | --- | --- | --- | --- | --- | --- | --- | --- | --- | --- | --- | --- | --- | --- | --- | --- |
|  |  | **1:10** | | | **1:100** | | | **1:1000** | | | **1:1500** | | | **1:2000** | | |
|  |  | **Mean OD ± SD (+)** | **Mean OD ± SD (-)** | **P/N ratio** | **Mean OD ± SD (+)** | **Mean OD ± SD (-)** | **P/N ratio** | **Mean OD ± SD (+)** | **Mean OD ± SD (-)** | **P/N ratio** | **Mean OD ± SD (+)** | **Mean OD ± SD (-)** | **P/N ratio** | **Mean OD ± SD (+)** | **Mean OD ± SD (-)** | **P/N ratio** |
| 1 | 5:1 | 0.316 ± 0.003 | 0.200 ± 0.001 | 1.582 | 0.327 ± 0.003 | 0.197 ± 0.002 | 1.659 | 0.336 ± 0.005 | 0.196 ± 0.001 | 1.711 | 0.314 ± 0.006 | 0.200 ± 0.002 | 1.566 | 0.275 ± 0.039 | 0.190 ± 0.002 | 1.447 |
|  | 10:1 | 0.319 ± 0.004 | 0.201 ± 0.002 | 1.586 | 0.327 ± 0.003 | 0.200 ± 0.001 | 1.635 | 0.343 ± 0.002 | 0.197 ± 0.002 | 1.738 | 0.326 ± 0.003 | 0.200 ± 0.003 | 1.629 | 0.304 ± 0.004 | 0.192 ± 0.002 | 1.584 |
|  | 20:1 | 0.326 ± 0.003 | 0.197 ± 0.002 | 1.650 | 0.329 ± 0.006 | 0.196 ± 0.001 | 1.683 | 0.370 ± 0.002 | 0.193 ± 0.003 | 1.919 | 0.334 ± 0.004 | 0.198 ± 0.003 | 1.685 | 0.326 ± 0.005 | 0.190 ± 0.002 | 1.714 |
|  | 30:1 | 0.325 ± 0.004 | 0.218 ± 0.004 | 1.495 | 0.337 ± 0.005 | 0.218 ± 0.003 | 1.544 | 0.346 ± 0.005 | 0.200 ± 0.001 | 1.727 | 0.338 ± 0.004 | 0.204 ± 0.006 | 1.661 | 0.332 ± 0.003 | 0.195 ± 0.003 | 1.701 |
|  | 40:1 | 0.325 ± 0.003 | 0.223 ± 0.001 | 1.454 | 0.334 ± 0.002 | 0.221 ± 0.003 | 1.515 | 0.345 ± 0.002 | 0.210 ± 0.001 | 1.643 | 0.336 ± 0.003 | 0.202 ± 0.003 | 1.663 | 0.328 ± 0.002 | 0.200 ± 0.001 | 1.641 |
|  | 50:1 | 0.332 ± 0.002 | 0.241 ± 0.002 | 1.376 | 0.342 ± 0.003 | 0.232 ± 0.003 | 1.474 | 0.350 ± 0.001 | 0.221 ± 0.002 | 1.580 | 0.343 ± 0.005 | 0.211 ± 0.002 | 1.627 | 0.335 ± 0.004 | 0.208 ± 0.003 | 1.612 |

| **Capture antibody (µg/well)** | **Biotin-labeled 4D1 (ratio)** | **Detection biotin-labeled 4D1 dilution** | | | | | | | | | | | | | | |
| --- | --- | --- | --- | --- | --- | --- | --- | --- | --- | --- | --- | --- | --- | --- | --- | --- |
|  |  | **1:10** | | | **1:100** | | | **1:1000** | | | **1:1500** | | | **1:2000** | | |
|  |  | **Mean OD ± SD (+)** | **Mean OD ± SD (-)** | **P/N ratio** | **Mean OD ± SD (+)** | **Mean OD ± SD (-)** | **P/N ratio** | **Mean OD ± SD (+)** | **Mean OD ± SD (-)** | **P/N ratio** | **Mean OD ± SD (+)** | **Mean OD ± SD (-)** | **P/N ratio** | **Mean OD ± SD (+)** | **Mean OD ± SD (-)** | **P/N ratio** |
| 2.5 | 5:1 | 0.415 ± 0.005 | 0.212 ± 0.002 | 1.958 | 0.412 ± 0.005 | 0.210 ± 0.001 | 1.965 | 0.406 ± 0.005 | 0.197 ± 0.002 | 2.061 | 0.399 ± 0.001 | 0.185 ± 0.004 | 2.159 | 0.379 ± 0.001 | 0.178 ± 0.002 | 2.123 |
|  | 10:1 | 0.425 ± 0.002 | 0.218 ± 0.004 | 1.950 | 0.420 ± 0.002 | 0.212 ± 0.002 | 1.984 | 0.422 ± 0.002 | 0.209 ± 0.002 | 2.014 | 0.410 ± 0.002 | 0.196 ± 0.002 | 2.090 | 0.400 ± 0.001 | 0.192 ± 0.003 | 2.083 |
|  | 20:1 | 0.589 ± 0.001 | 0.223 ± 0.002 | 2.643 | 0.589 ± 0.001 | 0.220 ± 0.001 | 2.677 | 0.611 ± 0.002 | 0.198 ± 0.001 | 3.084 | 0.571 ± 0.006 | 0.190 ± 0.002 | 2.998 | 0.473 ± 0.016 | 0.183 ± 0.004 | 2.585 |
|  | 30:1 | 0.591 ± 0.001 | 0.225 ± 0.001 | 2.621 | 0.588 ± 0.008 | 0.222 ± 0.001 | 2.646 | 0.593 ± 0.011 | 0.206 ± 0.003 | 2.874 | 0.544 ± 0.005 | 0.200 ± 0.001 | 2.722 | 0.536 ± 0.004 | 0.195 ± 0.003 | 2.752 |
|  | 40:1 | 0.599 ± 0.002 | 0.232 ± 0.003 | 2.583 | 0.592 ± 0.011 | 0.230 ± 0.001 | 2.572 | 0.606 ± 0.008 | 0.208 ± 0.004 | 2.920 | 0.569 ± 0.016 | 0.203 ± 0.006 | 2.803 | 0.538 ± 0.005 | 0.205 ± 0.005 | 2.627 |
|  | 50:1 | 0.601 ± 0.001 | 0.236 ± 0.002 | 2.543 | 0.593 ± 0.006 | 0.232 ± 0.002 | 2.556 | 0.602 ± 0.005 | 0.210 ± 0.001 | 2.873 | 0.550 ± 0.006 | 0.209 ± 0.001 | 2.633 | 0.536 ± 0.003 | 0.203 ± 0.006 | 2.636 |

| **Capture antibody (µg/well)** | **Biotin-labeled 4D1 (ratio)** | **Detection biotin-labeled 4D1 dilution** | | | | | | | | | | | | | | |
| --- | --- | --- | --- | --- | --- | --- | --- | --- | --- | --- | --- | --- | --- | --- | --- | --- |
|  |  | **1:10** | | | **1:100** | | | **1:1000** | | | **1:1500** | | | **1:2000** | | |
|  |  | **Mean OD ± SD (+)** | **Mean OD ± SD (-)** | **P/N ratio** | **Mean OD ± SD (+)** | **Mean OD ± SD (-)** | **P/N ratio** | **Mean OD ± SD (+)** | **Mean OD ± SD (-)** | **P/N ratio** | **Mean OD ± SD (+)** | **Mean OD ± SD (-)** | **P/N ratio** | **Mean OD ± SD (+)** | **Mean OD ± SD (-)** | **P/N ratio** |
| **5** | 5:1 | 0.717 ± 0.004 | 0.205 ± 0.004 | 3.490 | 0.705 ± 0.006 | 0.206 ± 0.001 | 3.426 | 0.696 ± 0.006 | 0.198 ± 0.001 | 3.509 | 0.681 ± 0.001 | 0.188 ± 0.002 | 3.618 | 0.603 ± 0.001 | 0.180 ± 0.002 | 3.342 |
|  | 10:1 | 0.729 ± 0.006 | 0.219 ± 0.002 | 3.332 | 0.713 ± 0.007 | 0.211 ± 0.002 | 3.375 | 0.704 ± 0.006 | 0.208 ± 0.002 | 3.383 | 0.700 ± 0.002 | 0.196 ± 0.001 | 3.576 | 0.652 ± 0.002 | 0.191 ± 0.001 | 3.420 |
|  | **20:1** | 1.231 ± 0.004 | 0.228 ± 0.003 | 5.399 | 1.223 ± 0.005 | 0.222 ± 0.003 | 5.511 | **1.315 ± 0.004** | **0.177 ± 0.009** | **7.443** | 1.145 ± 0.001 | 0.178 ± 0.008 | 6.443 | 1.036 ± 0.007 | 0.169 ± 0.001 | 6.128 |
|  | 30:1 | 1.238 ± 0.006 | 0.256 ± 0.002 | 4.831 | 1.218 ± 0.006 | 0.242 ± 0.001 | 5.028 | 1.220 ± 0.006 | 0.233 ± 0.003 | 5.235 | 1.101 ± 0.001 | 0.222 ± 0.004 | 4.954 | 1.003 ± 0.016 | 0.218 ± 0.003 | 4.608 |
|  | 40:1 | 1.221 ± 0.002 | 0.255 ± 0.005 | 4.782 | 1.219 ± 0.001 | 0.245 ± 0.004 | 4.974 | 1.197 ± 0.007 | 0.242 ± 0.003 | 4.953 | 1.107 ± 0.004 | 0.221 ± 0.015 | 5.017 | 1.000 ± 0.002 | 0.222 ± 0.002 | 4.511 |
|  | 50:1 | 1.232 ± 0.009 | 0.307 ± 0.006 | 4.016 | 1.217 ± 0.005 | 0.256 ± 0.003 | 4.761 | 1.212 ± 0.009 | 0.254 ± 0.004 | 4.764 | 1.102 ± 0.001 | 0.238 ± 0.001 | 4.622 | 1.001 ± 0.002 | 0.235 ± 0.003 | 4.254 |

| **Capture antibody (µg/well)** | **Biotin-labeled 4D1 (ratio)** | **Detection biotin-labeled 4D1 dilution** | | | | | | | | | | | | | | |
| --- | --- | --- | --- | --- | --- | --- | --- | --- | --- | --- | --- | --- | --- | --- | --- | --- |
|  |  | **1:10** | | | **1:100** | | | **1:1000** | | | **1:1500** | | | **1:2000** | | |
|  |  | **Mean OD ± SD (+)** | **Mean OD ± SD (-)** | **P/N ratio** | **Mean OD ± SD (+)** | **Mean OD ± SD (-)** | **P/N ratio** | **Mean OD ± SD (+)** | **Mean OD ± SD (-)** | **P/N ratio** | **Mean OD ± SD (+)** | **Mean OD ± SD (-)** | **P/N ratio** | **Mean OD ± SD (+)** | **Mean OD ± SD (-)** | **P/N ratio** |
| 7.5 | 5:1 | 0.707 ± 0.004 | 0.238 ± 0.004 | 2.965 | 0.698 ± 0.002 | 0.224 ± 0.003 | 3.118 | 0.693 ± 0.006 | 0.218 ± 0.002 | 3.177 | 0.681 ± 0.001 | 0.209 ± 0.001 | 3.253 | 0.652 ± 0.002 | 0.207 ± 0.010 | 3.143 |
|  | 10:1 | 0.711 ± 0.001 | 0.242 ± 0.001 | 2.942 | 0.697 ± 0.007 | 0.232 ± 0.002 | 3.007 | 0.695 ± 0.005 | 0.221 ± 0.003 | 3.140 | 0.690 ± 0.001 | 0.219 ± 0.001 | 3.152 | 0.674 ± 0.005 | 0.212 ± 0.002 | 3.181 |
|  | 20:1 | 1.121 ± 0.002 | 0.254 ± 0.003 | 4.418 | 1.109 ± 0.001 | 0.242 ± 0.001 | 4.590 | 1.010 ± 0.002 | 0.232 ± 0.001 | 4.360 | 0.958 ± 0.005 | 0.223 ± 0.002 | 4.290 | 0.949 ± 0.001 | 0.217 ± 0.007 | 4.738 |
|  | 30:1 | 1.187 ± 0.009 | 0.286 ± 0.007 | 4.154 | 1.107 ± 0.008 | 0.266 ± 0.001 | 4.158 | 1.004 ± 0.005 | 0.258 ± 0.003 | 3.895 | 1.001 ± 0.001 | 0.243 ± 0.002 | 4.121 | 0.985 ± 0.004 | 0.227 ± 0.004 | 4.331 |
|  | 40:1 | 1.201 ± 0.002 | 0.316 ± 0.006 | 3.797 | 1.187 ± 0.032 | 0.299 ± 0.001 | 3.973 | 1.117 ± 0.021 | 0.286 ± 0.004 | 3.909 | 1.001 ± 0.005 | 0.275 ± 0.002 | 3.650 | 0.990 ± 0.001 | 0.261 ± 0.003 | 3.799 |
|  | 50:1 | 1.210 ± 0.008 | 0.351 ± 0.001 | 3.450 | 1.203 ± 0.005 | 0.341 ± 0.003 | 3.525 | 1.180 ± 0.001 | 0.312 ± 0.001 | 3.782 | 1.112 ± 0.005 | 0.304 ± 0.005 | 3.658 | 1.109 ± 0.001 | 0.287 ± 0.003 | 3.858 |

| **Capture antibody (µg/well)** | **Biotin-labeled 4D1 (ratio)** | **Detection biotin-labeled 4D1 dilution** | | | | | | | | | | | | | | |
| --- | --- | --- | --- | --- | --- | --- | --- | --- | --- | --- | --- | --- | --- | --- | --- | --- |
|  |  | **1:10** | | | **1:100** | | | **1:1000** | | | **1:1500** | | | **1:2000** | | |
|  |  | **Mean OD ± SD (+)** | **Mean OD ± SD (-)** | **P/N ratio** | **Mean OD ± SD (+)** | **Mean OD ± SD (-)** | **P/N ratio** | **Mean OD ± SD (+)** | **Mean OD ± SD (-)** | **P/N ratio** | **Mean OD ± SD (+)** | **Mean OD ± SD (-)** | **P/N ratio** | **Mean OD ± SD (+)** | **Mean OD ± SD (-)** | **P/N ratio** |
| 10 | 5:1 | 0.707 ± 0.004 | 0.243 ± 0.002 | 2.912 | 0.698 ± 0.002 | 0.233 ± 0.003 | 2.993 | 0.693 ± 0.006 | 0.224 ± 0.003 | 3.088 | 0.681 ± 0.001 | 0.210 ± 0.002 | 3.238 | 0.652 ± 0.002 | 0.207 ± 0.004 | 3.148 |
|  | 10:1 | 0.711 ± 0.001 | 0.250 ± 0.001 | 2.846 | 0.697 ± 0.007 | 0.241 ± 0.002 | 2.887 | 0.695 ± 0.005 | 0.222 ± 0.002 | 3.135 | 0.690 ± 0.001 | 0.221 ± 0.002 | 3.124 | 0.674 ± 0.005 | 0.212 ± 0.003 | 3.181 |
|  | 20:1 | 1.121 ± 0.002 | 0.257 ± 0.002 | 4.355 | 1.109 ± 0.001 | 0.247 ± 0.005 | 4.497 | 1.010 ± 0.002 | 0.235 ± 0.003 | 4.304 | 0.958 ±00 | 0.232 ± 0.003 | 4.135 | 0.949 ± 0.010 | 0.220 ± 0.005 | 4.319 |
|  | 30:1 | 1.187 ± 0.009 | 0.290 ± 0.002 | 4.092 | 1.107 ± 0.008 | 0.272 ± 0.001 | 4.071 | 1.004 ± 0.005 | 0.261 ± 0.002 | 3.841 | 1.001 ± 0.001 | 0.246 ± 0.003 | 4.076 | 0.985 ± 0.004 | 0.230 ± 0.001 | 4.281 |
|  | 40:1 | 1.201 ± 0.002 | 0.321 ± 0.001 | 3.745 | 1.187 ± 0.032 | 0.311 ± 0.001 | 3.816 | 1.117 ± 0.021 | 0.295 ± 0.004 | 3.781 | 1.004 ± 0.005 | 0.279 ± 0.002 | 3.593 | 0.990 ± 0.001 | 0.262 ± 0.003 | 3.775 |
|  | 50:1 | 1.210 ± 0.008 | 0.353 ± 0.001 | 3.424 | 1.203 ± 0.005 | 0.342 ± 0.003 | 3.519 | 1.180 ± 0.001 | 0.323 ± 0.001 | 3.653 | 1.112 ± 0.005 | 0.308 ± 0.006 | 3.614 | 1.109 ± 0.001 | 0.288 ± 0.004 | 3.850 |

**Note:**

Consistent conditions:

TMCYA (10 µg/well)

Streptavidin-HRP (1:5000)

Variable conditions:

Capture antibody (0.1-10 µg/well)

Biotin-labeled 4D1 (5:1-50:1) (dilution 1:10-1:2000)

Data are presented as mean ± standard deviation (SD) from at least three independent experiments. Mean OD (+) denotes the optical density of positive samples (with TMCYA), whereas Mean OD (−) denotes the optical density of negative controls (without TMCYA).

The P/N ratio was calculated as the ratio of the mean OD value of positive samples to that of negative controls.

Blank OD values were low and consistent across all conditions (approximately 0.06-0.07).

The optimal condition is highlighted in bold.

**Table D. Optimization of streptavidin-HRP dilution in the sandwich ELISA**

| **Streptavidin–HRP dilution** | **Mean OD (+) ± SD (+)** | **Mean OD (−) ± SD (-)** | **P/N ratio** |
| --- | --- | --- | --- |
| 1:1000 | 1.433 ± 0.005 | 0.384 ± 0.003 | 3.734 |
| 1:2000 | 1.407 ± 0.006 | 0.302 ± 0.003 | 4.659 |
| 1:3000 | 1.397 ± 0.001 | 0.261 ± 0.002 | 5.354 |
| 1:4000 | 1.329 ± 0.002 | 0.234 ± 0.002 | 5.673 |
| 1:5000 | 1.325 ± 0.004 | 0.179 ± 0.001 | 7.388 |
| **1:6000** | **1.333 ± 0.005** | **0.172 ± 0.004** | **7.763** |
| 1:7000 | 1.006 ± 0.009 | 0.161 ± 0.001 | 6.251 |
| 1:8000 | 0.989 ± 0.002 | 0.154 ± 0.003 | 6.422 |

**Note:**

Consistent conditions:

TMCYA (10 µg/well)

Capture antibody (mAb 4D1, 5 µg/well)

Biotin-labeled 4D1 20:1 (dilution 1:1000)

Variable conditions:

Streptavidin-HRP dilution (1:1000-1:8000)

Data are presented as mean ± standard deviation (SD) from at least three independent experiments. Mean OD (+) represents the optical density of positive samples (with TMCYA), whereas Mean OD (−) represents that of negative controls (without TMCYA).

The P/N ratio was calculated as the ratio of the mean OD value of positive samples to that of negative controls.

The optimal condition is highlighted in bold.

**Table E. Optimization of serum dilution for antigen detection in the sandwich ELISA**

| **Serum dilution** | **Mean OD (+) ± SD (+)** | **Mean OD (−) ± SD (−)** | **P/N ratio** |
| --- | --- | --- | --- |
| 1:2 | 0.796 ± 0.006 | 0.307 ± 0.006 | 2.593 |
| 1:4 | 0.679 ± 0.002 | 0.257 ± 0.003 | 2.643 |
| 1:8 | 0.520 ± 0.001 | 0.225 ± 0.004 | 2.313 |
| **1:10** | **0.496 ± 0.006** | **0.154 ± 0.004** | **3.216** |
| 1:20 | 0.455 ± 0.005 | 0.170 ± 0.002 | 2.673 |
| 1:40 | 0.328 ± 0.006 | 0.167 ± 0.004 | 1.966 |
| 1:80 | 0.298 ± 0.001 | 0.168 ± 0.003 | 1.768 |
| 1:100 | 0.215 ± 0.002 | 0.166 ± 0.002 | 1.293 |

**Note:**

Consistent conditions:

TMCYA-spiked serum (initial concentration: 100 µg/mL)

Capture antibody (mAb 4D1, 5 µg/well)

Biotin-labeled 4D1 20:1 (dilution 1:1000)

Streptavidin-HRP (dilution 1:6000)

Variable conditions:

Serum dilution (1:2-1:100), 100 µL/well

Data are presented as mean ± standard deviation (SD) from at least three independent experiments. Mean OD (+) represents the optical density of positive samples (TMCYA-spiked serum), whereas Mean OD (−) represents that of negative controls (non-spiked serum).

The P/N ratio was calculated as the ratio of the mean OD value of positive samples to that of negative controls.

Blank OD values were low and consistent across all conditions.

The optimal condition is highlighted in bold.


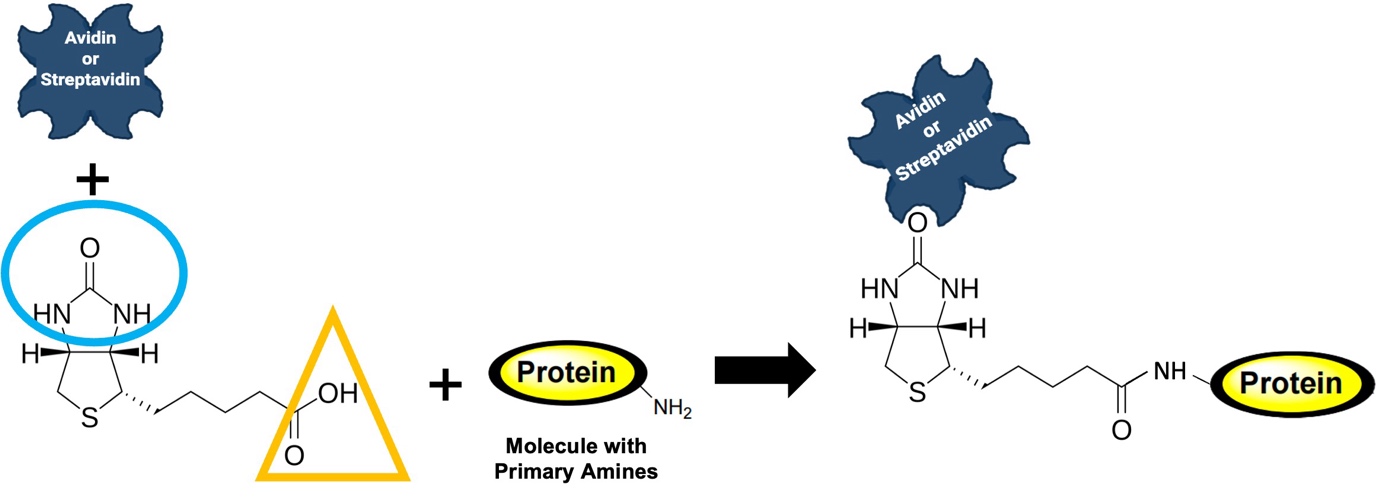


**Fig A.** **Biotinylation strategy showing NHS-ester activation and subsequent conjugation of avidin/streptavidin to primary amine-containing proteins.** The blue circle indicates the biotin moiety responsible for binding to avidin/streptavidin, whereas the orange triangle marks the site of conjugation to primary amine-containing proteins. The drawing is original and based on [43] modified with instructions from EZ-Link Sulfo-NHS-Biotinylation Kit (Thermo Fisher Scientific, Waltham, MA, USA).


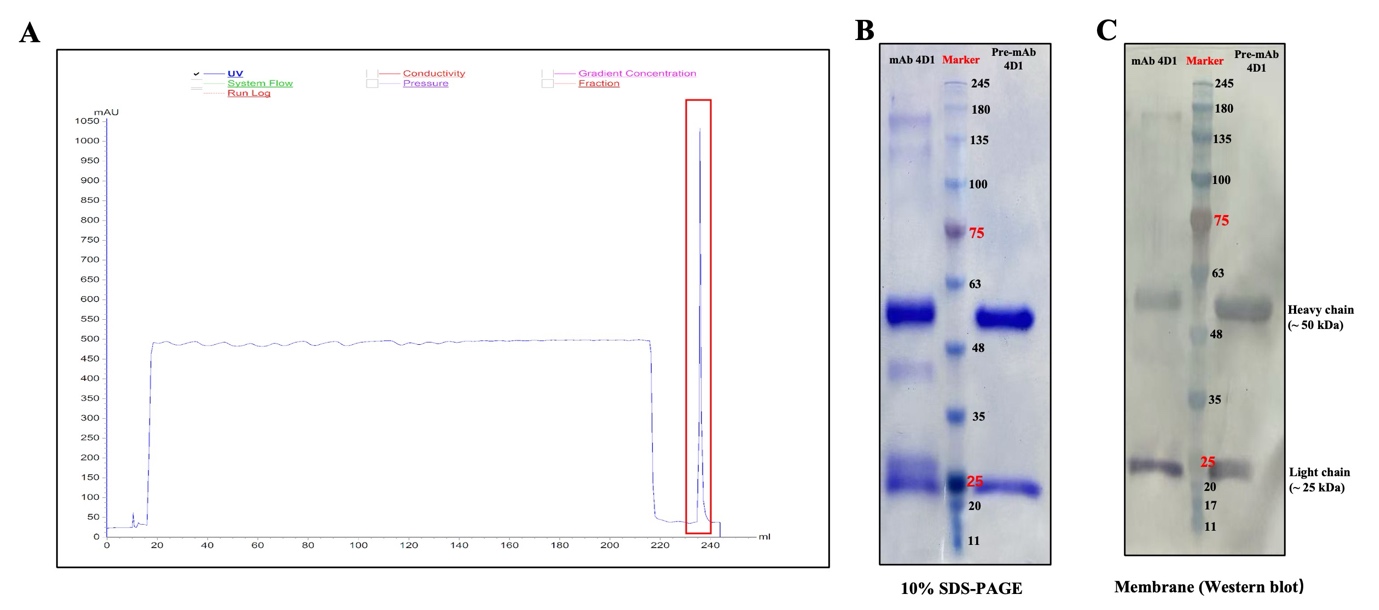


**Fig B.** **Purification of mAb 4D1. (A)** The diagram illustrates the UV absorbance profile during the purification of mAb 4D1 from hybridoma culture supernatant using an ÄKTA start chromatography system with a HiTrap protein G affinity column. The purity of the eluted fractions was assessed by monitoring the UV absorbance peak (in the red box). **(B)** SDS-PAGE analysis of purified mAb 4D1. The gel was run under reducing conditions (10 µg per lane). **(C)** Mouse IgG isotype determination of mAb 4D1 by Western blot using a goat anti-mouse IgG antibody (specific for the γ, κ, and λ chains). The pooled eluted fractions show both IgG subunits with molecular weights of 50 kDa (heavy chain) and 25 kDa (light chain). The positive control was a previous lot of mAb 4D1 described in Pruksaphon et al., 2018.





**Fig C.** **Controls validating the specificity of mAb 4D1 and its biotin-labeled conjugate in Western blot detection of TMCYA.** (A) Western blot probed with unconjugated mAb 4D1 followed by HRP-conjugated goat anti-mouse IgG secondary antibody, demonstrating a specific immunoreactive band exclusively in the TMCYA lane (lane 2) spanning approximately 50-150 kDa, with no reactivity detected against cytoplasmic antigens from any other fungal species tested. (B) Negative control for non-specific secondary antibody binding: membrane incubated with HRP-conjugated goat anti-mouse IgG in the absence of primary mAb 4D1, showing no detectable signal in any lane, confirming that background signal does not arise from non-specific secondary antibody binding. (C) Negative control for non-specific streptavidin binding: membrane incubated with streptavidin-HRP in the absence of biotin-labeled 4D1, showing no detectable signal in any lane, confirming that the positive signal observed with the biotin-4D1 conjugate is not attributable to endogenous biotin or non-specific streptavidin interactions. Lane assignments are as follows: lane 1, molecular weight marker; lane 2, *T. marneffei* yeast; lane 3, *T. marneffei* conidia; lane 4, *T. marneffei* mold; lane 5, *C. albicans*; lane 6, *Pichia kudriazevii*; lane 7, *Geotrichum* *sp*.; lane 8, *S. apiospermum*; lane 9, *Trichosporon* *sp.*; lane 10, *S. schenckii* (yeast); lane 11, *C. neoformans*; lane 12, *P. citrinum*; lane 13, A. fumigatus.





**Fig D. Optimization of capture and detection antibody conditions for the sandwich ELISA.** A range of coating antibody concentrations and biotin-labeled antibody dilutions were tested to determine the optimal antibody pairing. Signal intensity was evaluated using the difference between the mean OD values of positive and negative samples (ΔOD).

The optimal combination, corresponding to the maximum ΔOD value, was selected as it provided the best discrimination between positive and negative signals. This condition is highlighted by the red arrow. Each point represents the mean of replicate measurements.





**Fig E. Optimization of streptavidin-HRP dilution in the sandwich ELISA.** Following the determination of the optimal coating antibody concentration (mAb 4D1, 5 µg/well) and the optimal biotin-labeled antibody dilution, a series of streptavidin-HRP dilutions (1:1000-1:8000) were evaluated. Signal intensity was assessed using the difference between the mean OD values of positive samples and negative controls (ΔOD = Mean OD (+) − Mean OD (−)). The optimal streptavidin-HRP dilution was selected based on the maximum ΔOD value, indicating the greatest separation between positive and negative signals. The selected condition is indicated by the red arrow. Each point represents the mean of replicate measurements.





**Fig F. Optimization of serum dilution for antigen detection in the sandwich ELISA**. After determining the optimal coating antibody concentration (mAb 4D1, 5 µg/well), biotin-labeled antibody dilution, and streptavidin-HRP dilution, a series of serum dilutions (1:2-1:100) were evaluated. Signal intensity was assessed using the difference between the mean OD values of positive samples and negative controls (ΔOD = Mean OD (+) − Mean OD (−)). The optimal serum dilution was selected based on the maximum ΔOD value, indicating the greatest separation between positive and negative signals. The selected condition is indicated by the red arrow.





**Fig G. Determination of analytical performance parameters (LOB, LOD, and LOQ) of the biotin-labeled 4D1 sandwich ELISA.** Scatter plots represent OD450 values of blank pooled serum and TMCYA-spiked samples at increasing concentrations. The limit of blank (LOB) was calculated as the mean OD of blank samples plus 1.645 × standard deviation (SD). The limit of detection (LOD) was defined as the LOB plus 1.645 × SD of low-concentration samples. The OD value corresponding to the LOD was converted into antigen concentration using the four-parameter logistic (4-PL) standard curve, yielding an LOD of 19.398 μg/mL. The limit of quantification (LOQ) was defined as the lowest concentration that could be quantified with acceptable precision (CV < 20%) and was determined to be 25 μg/mL. Horizontal dashed lines indicate the LOB, LOD, and LOQ thresholds.
