# Supplementary material for: Diagnostic performance of a biotin-labeled 4D1 sandwich ELISA for serum antigen detection in talaromycosis
Source: PLoS Negl Trop Dis. 2026 May 5;20(5):e0014314. doi: 10.1371/journal.pntd.0014314 (PMC13167033; doi:10.1371/journal.pntd.0014314)
Supplement: Attachment — Submitted filename: 3.Response--Reviewer 3.docx [file pntd.0014314.s003.docx]

**Response to reviewer #3 comments**

We sincerely appreciate your time and effort in reviewing our manuscript, as well as your insightful comments and constructive suggestions. All the points raised have been carefully evaluated and thoroughly addressed. A detailed, point-by-point response is provided below. Corresponding revisions have been incorporated into the manuscript to reflect our responses and improvements.

**Methods:**

| **No.** | **Reviewer comment** | **Author answer** |
| --- | --- | --- |
| **1** | The methodology is very clear, and the hypothesis and objectives are well defined; I think we managed to cover the proposed topic well. | We thank the reviewer for the positive and encouraging comments. We are pleased that the methodology, hypothesis, and objectives of the study are considered clear and well defined, and that the manuscript is regarded as having adequately addressed the proposed topic. No further changes were required in response to this comment. |

**Results:**

| **No.** | **Reviewer comment** | **Author answer** |
| --- | --- | --- |
| **1** | The results were consistent with the proposal, although some data were presented in a confusing manner; more detailed comments follow below:  Supplementary Figure 3 (A, B, and C) is confusing and difficult to understand what is being shown. Perhaps the quality could be improved, and the bands and sizes of interest could be indicated. | We thank the reviewer for this helpful and constructive comment. We agree that the original presentation of Supplementary Figure 3 (A-C) may have been unclear.In response, we have improved the image quality by optimizing contrast and resolution to enhance band visibility. The bands corresponding to the target antigen (TMCYA, approximately 50-150 kDa) are now clearly indicated with arrows. In addition, molecular weight markers (kDa) have been included in each panel to facilitate interpretation.Furthermore, lane labeling has been clarified, and the figure legend has been revised to provide a more detailed and accurate description of the experimental setup and results. These revisions significantly improve the clarity and interpretability of the figure. |
| **2** | The expected sizes in the gel in Figure 3 could also be better indicated. | We thank the reviewer for this helpful comment. We agree that the expected molecular sizes in Figure 3 could be more clearly indicated.  In response, we have revised Figure 3 to improve clarity by adding molecular weight markers (kDa) to the gel. In addition, the expected size range of the target antigen (approximately 50-150 kDa) is now clearly indicated using annotations and arrows. These revisions enhance the interpretability of the gel and facilitate easier identification of the bands of interest.  These changes have been incorporated into the revised manuscript (Page 17-18, Lines 422-437). |
| **3** | The table 3 is confusing; I suggest inverting it, showing how many of the positive and negative (total) included in the study were analyzed, and then showing the results of the biotin-labeled 4D1 sandwich ELISA. | We thank the reviewer for this helpful suggestion. We agree that the original presentation of Table 3 may have been less intuitive.  In response, we have revised the table to present the data according to culture status (reference standard), including the total number of positive and negative cases analyzed, followed by the corresponding results of the biotin-labeled 4D1 sandwich ELISA. This revised format more clearly reflects the diagnostic performance and improves overall readability.  The updated table has been incorporated into the revised manuscript (Page 22-23, Lines 534-538). |

**Conclusions:**

| **No.** | **Reviewer comment** | **Author answer** |
| --- | --- | --- |
| **1** | Yes, the topic is relevant and I believe it opens doors to new diagnostic possibilities and fills an important gap. The limitations were well explored in the discussion. | We sincerely thank the reviewer for this positive and encouraging comment. We are grateful for the recognition of the relevance of our work and its potential contribution to addressing an important gap in the field. We also appreciate the reviewer’s acknowledgment of the discussion of the study limitations. |

**Editorial and Data Presentation Modifications?**

| **No.** | **Reviewer comment** | **Author answer** |
| --- | --- | --- |
| **1** | Suggestions: "292 The second membrane was incubated with biotin-labeled 4D1 (20:1, diluted 1:1000 in 1% (*w/v*) BSA prepared in 10 mM Tris-HCl, pH 7.4) for 2 hours and subsequently incubated with streptavidin-HRP diluted 1:6000 in 1% (*w/v*) BSA prepared in 10 mM Tris-HCl (pH 7.4) for 1 hour." Explain how you arrived at this dilution of biotin-labeled 4D1. | We thank the reviewer for this valuable and constructive comment.  The dilution of key assay components, including biotin-labeled 4D1, streptavidin-HRP, and serum samples, was determined through systematic preliminary optimization using serial dilutions. Specifically, a range of dilution ratios was evaluated to identify conditions that provided the optimal signal-to-noise ratio while minimizing background interference.  The selected conditions (biotin-labeled 4D1 at 1:1000, streptavidin-HRP at 1:6000, and serum dilution at 1:10) yielded the best balance between signal intensity and specificity.  To address the reviewer’s concern, the corresponding optimization data have now been included in the Supplementary Materials (Supplementary Tables 3-5 and Supplementary Figures 4-6 ), comprising representative figures and summary tables that illustrate the systematic evaluation process and support the selection of these working conditions.  In addition, a brief description of the optimization strategy has been incorporated into the Methods section (Page 13-14, Lines 323-335) to clarify the rationale for the selected dilutions. |
| **2** | 524 "Moreover, the assay exhibited no cross-reactivity wit common fungal, bacterial, or viral antigens, demonstrating its strong potential for clinical application"  No bacterial or viral data was shown here; therefore, add a reference that shows this information. | We thank the reviewer for this important comment. We agree that cross-reactivity with bacterial and viral antigens was not evaluated in the present study.  In response, we have revised the sentence to accurately reflect our experimental data by limiting the statement to the tested fungal antigens only and removing unsupported claims. Therefore, no additional references were included, as the revised statement is now strictly based on the results obtained in this study.  The revised text now reads:  “In this study, the assay exhibited no cross-reactivity with the tested fungal antigens, supporting its specificity for *T. marneffei* and highlighting its potential for clinical application.”（Page 25, Lines 589-591）  This modification improves the accuracy of the statement and avoids overinterpretation of the results. |

We hope that these revisions have adequately addressed the reviewer’s comments and have improved the clarity and overall quality of the manuscript. We sincerely appreciate the reviewer’s constructive feedback, which has greatly strengthened our work.
